# Women’s and communities’ views of targeted educational interventions to reduce unnecessary caesarean section: a qualitative evidence synthesis

**DOI:** 10.1186/s12978-018-0570-z

**Published:** 2018-07-24

**Authors:** Carol Kingdon, Soo Downe, Ana Pilar Betran

**Affiliations:** 1 School of Community Health and Midwifery, Faculty of Health and Wellbeing, University of Central Lancashire, Preston, PR1 2HE UK; 2 Department of Reproductive Health and Research, World Health Organization (WHO), Geneva, Switzerland

**Keywords:** Caesarean section, Educational interventions, Decision-aids, Birth method, Women’s views

## Abstract

**Background:**

There is continued debate about the role of women and communities in influencing rising rates of caesarean section (CS). In settings where CS rates exceed recommended levels, mothers and babies are exposed to potential harms that may outweigh the potential benefits. There is therefore a need to understand how educational interventions targeted at women and communities to reduce unnecessary CS are perceived and used. This qualitative evidence synthesis aimed to explore what women and communities say about the barriers and facilitators to intervention effectiveness for these important groups.

**Method:**

Seven electronic databases were searched using predefined search terms. Studies reporting qualitative data pertaining to interventions, published between 1985 and March 2017, with no language restriction were sought. Study quality was independently assessed by two authors before qualitative evidence synthesis was undertaken using an interpretive, meta-ethnography approach. Resulting Statements of Findings were assessed using GRADE-CERQual, and summarised thematically.

**Results:**

Twelve studies were included. They were published between 2001 and 2016. Eleven were from high-income countries. Twelve Summaries of Findings encompassed the data, and were graded (moderate or high) on CerQual. The Statements of Findings are reported under three final themes: 1) Mutability of women’s and communities’ beliefs about birth; 2) Multiplicity of individual information needs about birth; 3) Interactions with health professionals and influence of healthcare system on actual birth method. Women and communities value educational interventions that include opportunities for dialogue, are individualised (including acknowledgement of previous birth experiences), and are consistent with available clinical care and the advice of the health professional they come into contact with.

**Conclusion:**

Women’s values and preferences for birth, and for information format and content, vary across populations, and evolves in individual women over time. Interactions with health professionals and health system factors can partly be responsible for changes in views. Educational interventions should take into account these dynamic interactions, as well as the women’s need for emotional support and dialogue with professionals alongside information about birth. Further research is required to test these findings and the utility of their practical application, particularly in medium and low income settings.

**Systematic review registration number:**

PROSPERO 2017 CRD42017059453.

**Electronic supplementary material:**

The online version of this article (10.1186/s12978-018-0570-z) contains supplementary material, which is available to authorized users.

## Plain English summary

### Why did we do this review?

Caesarean section (CS) can be life-saving in some circumstances. However, current rates suggest that the operation is sometimes used for healthy women and babies when it is not medically necessary. Reasons include health service factors, clinician convenience, and women’s choice. If a caesarean section is not medically needed, the benefits can be outweighed by the risks of harm. This is now a global concern. It is not yet clear how the views and experiences of women and of communities are affected by what they know about the possible risks and benefits of CS. We particularly wanted to know what people say about educational interventions designed to safely reduce CS rates.

### What did we find?

We identified 12 studies, published between 2001 and 2016. Eleven were from high-income countries (USA, Norway, Australia, Canada, UK, Taiwan). One was from a middle-income country (Brazil). Seven involved women who had had a previous CS. We reviewed the studies using qualitative evidence synthesis methods. We found women and communities value educational interventions that include opportunities for dialogue, are individualised (including acknowledgement of previous birth experiences), and are consistent with available clinical care and the advice of health professionals they encounter. We have more confidence in these results for women and communities in high-income countries because of where most of the contributing studies were conducted. Future educational interventions in high income countries should be based on these results, and further research is needed to find out if these interventions are also relevant for middle and low income countries.

## Background

When medically indicated, caesarean section (CS) can prevent deaths and other serious complications in mothers and babies. However, there is evidence of risks for some healthy women and babies undergoing CS [1–3]. The World Health Organization’s (WHO) 2015 Statement declares that rates higher than 10% are not associated with reductions in maternal and newborn mortality, and can cause surgical complications, disability or death, particularly in settings that lack the facilities and/or capacity to properly conduct safe surgery [3]. Several countries now have national CS rates above 50%. The average global CS rate is 18.6%, ranging from 6.0 to 27.2% in the least and the more developed regions [4]. In any setting, women with term pregnancies, with a single fetus and a cephalic presentation (Robson Groups 1–5) are the main contributors to rates representing between 75 and 80% of all CSs conducted [5].

Since 2015, global concern about unnecessary CS has increased [3, 6, 7]. Data from 2008 suggest 6.2 million women undergo unnecessary CS annually, at an estimated cost of 2.32 billion US dollars [8]. Despite the global concern and parallel research conducted, effective interventions tested to reduce unnecessary CS have been elusive [9]. The reason for this limited success may lie in the multifactorial nature of the increase and the multiple stakeholders involved. Women, healthcare professionals, systems, culture and society are all key players, whose concerns contribute to the current situation. There is evidence from across settings that women have become more active in seeking CS [10–16]. One systematic review suggests higher preference for CS amongst women with a previous CS, and those living in middle-, versus high-income countries [17]. Non-clinical, educational interventions targeted at women have been proposed and tested to reduce unnecessary CS. These interventions include workshops, booklets, decision-aids [9]. Qualitative evidence of how these interventions are perceived and used is a missing, but essential component to inform the design of future strategies targeting women. The aim of this synthesis was to provide new evidence of what women, communities and publics say about the success or failure of educational interventions targeted at them to reduce unnecessary CS, including barriers and facilitators to intervention use.

## Method

This qualitative evidence synthesis used an interpretive meta-ethnography approach [18]. The funder had no role in the conduct of the research. The protocol was published by PROSPERO [19]. A PRISMA checklist [20] is provided as additional information (Additional file 1: Appendix S0).

### Searches

#### Electronic search strategies

Systematic search strategies were developed building on preliminary scoping searches, terms used by existing quantitative reviews of interventions to reduce unnecessary CS, [21–23] guidelines developed by the Cochrane Qualitative Research Methods Group, [24–27] and papers detailing strategies for optimising the identification of qualitative studies in CINAHL, [26] MEDINE [27], EMBASE [28] and PsycINFO [29]. An example search strategy is provided as additional information (Additional file 2: Appendix S1). CINAHL, MEDLINE, PsycINFO, EMBASE, Global Index Medicus, POPLINE, and African Journals Online were searched for eligible studies published between 1st January 1985 and the date of final search (22nd March 2017), to identify studies since the first WHO statement on appropriate technology for childbirth [30]. We imposed no language or geographic restrictions.

#### Other sources

**Fig. 1:**
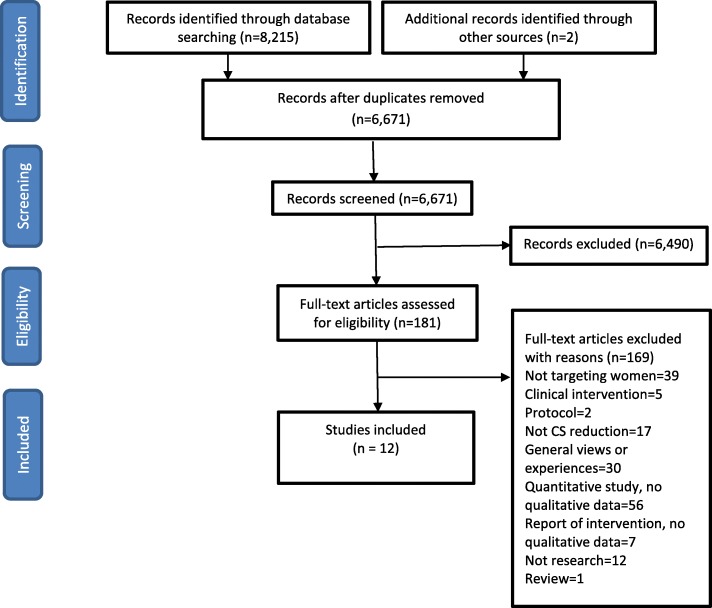
PRISMA flow diagram

As retrieval of qualitative research using databases alone is limited, the reference lists of all the included studies and existing quantitative reviews were back and citation chained [21–23]. In addition, key articles cited by multiple authors (citation pearls) were checked on Google Scholar. The authors of published protocols were also contacted [31, 32]. See Fig. 1 (PRISMA Flow Diagram) for an overview of the study identification, screening eligibility and inclusion process.

### Criteria for inclusion and exclusion of studies

#### Types of study designs

All studies utilising a qualitative research design (e.g. ethnography, phenomenology), or qualitative methods for data collection (e.g. focus group interviews, individual interviews, observation, diaries, oral histories), and which used qualitative methods for data analysis (e.g. thematic analysis, framework approach, grounded theory, thematic network analysis) were eligible for inclusion. Studies using mixed methods designs were also eligible where it was possible to extract findings derived from the qualitative component. Studies in which data were collected using qualitative methods, but which did not perform a qualitative analysis (e.g. if qualitative data were only reported using descriptive statistics), were excluded.

#### Types of participants

The population of interest was women in general (defined as any woman of reproductive age; pregnant or non-pregnant) and for pregnant women, those that could be considered low risk (i.e. we excluded studies that included only women with multiple pregnancies, or breeches or where the fetal lie was transverse or oblique) but we included women with previous CS. The Robson 10-Group classification (see Additional file 3: Figure S1) were used as an approximation to illustrate the types of women covered by this synthesis because it is an internationally accepted classification and provides a useful framework for action [33, 34]. Women in Robson Groups 1–5 are the main contributors to high CS rates in any setting and likely the groups where more unnecessary CSs concentrate [5].

#### Types of intervention

For the purposes of this synthesis an intervention was ‘anything considered by study authors as an intervention additional to usual care undertaken with the aim of reducing unnecessary caesarean section’ [19]. This definition was purposively broad to encompass known and unknown interventions [22]. Interventions targeted at women with a breech presentation (Robson Groups 6 and 7), multiple pregnancy (Robson Group 8), transverse or oblique lie (Robson Group 9) or preterm birth (Robson Group 10) were excluded.

Identified electronic records were collated into one database and duplicates removed. Two synthesis authors (CK, SD) independently assessed each abstract to determine eligibility for inclusion against a priori inclusion and exclusion criteria. The full texts of all papers identified as potentially relevant were retrieved and also independently assessed by CK and SD. The view of the third author APB was sought before agreeing on the final list of included studies.

### Qualitative evidence synthesis

The meta-ethnography [18] approach to qualitative evidence synthesis approach used in this qualitative evidence synthesis comprised of five stages 1) Familiarisation and quality assessment, 2) Data extraction, 3) Coding, 4) Interpretative synthesis, 5) CERQual assessment [35]. For further detail see Additional file 4: Box S1: Qualitative Evidence Synthesis Methodology.

### Reflexivity statement

CK is a medical sociologist who held prior beliefs about the complexity and interdependency of social factors driving CS rates informed by primary research with women and health professionals in the UK. APB is a medical officer with over 15 years of experience in maternal and perinatal health research and public health and has witnessed the sense of helplessness and the barriers governments experience when trying to reduce unnecessary CS. SD, is a Professor of Midwifery, her interactions with the data were informed by her experience of the barriers clinical staff encounter on the ground when they try to use their clinical judgement and skills alongside personal values and knowledge of the current evidence base, and the views and choices of childbearing women.

## Results

Our electronic searches yielded 8215 citations. We screened 6671 unique records after duplicate removal. We assessed 181 full-text articles for eligibility and included 12 studies in this qualitative evidence synthesis. The included studies, published between 2001 and 2016, were from seven different countries and mostly from urban areas (Table 1) [36–48]. Samples ranged from 5 to 170 participants, consistent with the qualitative approached used. Eleven studies were from high-income settings (USA, Norway, Australia, Canada, UK, Taiwan). One study was from a middle-income setting (Brazil). Seven studies exclusively involved women with a previous CS (Robson Group 5), two were targeted at non-pregnant women and communities and three were in Robson Groups 1,2 and 5. Three of the twelve studies were qualitative [37, 39, 41] and sibling studies of trials [49, 50] included in the Cochrane Reviews of non-clinical interventions for reducing unnecessary CS [9, 22]. Quality assessment of studies ranged from A to C. Table 1 lists the quality assessment grades assigned. Seven studies were assessed as having no, few, or only some flaws, with credibility, transferability, dependability, and confirmability unlikely to have been affected (A-B). For each of the 12 studies, the intervention is also described in Table 1. Table 2 is the CERQual [35] summary of qualitative findings table. The development from coding of initial concepts to emergent themes into final themes [18] (with supporting data quotes), and the CERQual [35] summary of evidence profile are provided as (Additional file 5: Table S1; Additional file 6: Table S2). Hereafter, findings are reported under thematic headings with emergent theme sub-headings in bold.

**Table 1:** Summary of included studies and quality assessment

Author	Intervention	Country and region	Setting	Number of participants	Type of participants	Robson Groups	Method	Quality Assessment
McCants [47, 48]	Prebirth educational brochure.	USA, Americas	Urban	16	Non-pregnant women	Community^a^	In-depth interviews	C
Shorten [46]	Internet-based decision aid about birth choices after previous caesarean developed from an earlier paper-based decision aid.	USA, Americas	Urban	9	Pregnant women	5	Semi-structured Interviews	B
Ramvi [45]	Women who took part in a team Midwifery intervention referred because they requested a caesarean because of fear of birth and actually had a vaginal birth.	Norway, European	Urban	5	Postnatal women	1–5	In-depth interviews	A
Basso [44]	Problematizing educational intervention with groups of pregnant women and partners comprising eight face-to-face sessions.	Brazil, Americas	Urban	51	Pregnant women and partners	1 and 2	Convergent Care study	A
David [43]	Dedicated next birth after caesarean telephone helpline manned by midwives to promote safe and successful vaginal birth in a subsequent pregnancy.	Australia, Western Pacific	Unclear	170	Pregnant women	5	Content analysis	C
Milne [42]	Decision board comprising decisional aids that provide a standardized base of written and graphic evidence-based information about vaginal and caesarean birth.	Canada, Americas	Urban	40	Non-pregnant women	Community^a^	Semi-structured interviews	C
Frost [41]	Two computer decision aids (an information programme and an individualised decision analysis programme)	UK, European	Urban	30	Pregnant women	5	Semi-structured interviews	B
Farnworth [40]	Informational DVD/video and a home visit by a midwife	UK, European	Urban	18	Pregnant women	5	Semi-structured interview	B
Emmett [39]	Two decision aids (an information programme and an individualised decision analysis programme)	UK, European	Urban	26	Pregnant and postnatal women	5	Semi-structured interview and observation	B
Wang [38]	Web-based education program for vaginal birth after caesarean	Taiwan, Western Pacific	Urban	10	Postnatal women	5	Telephone interviews	C
Shorten [37]	Birth choices booklet: Development, preliminary evaluation and pilot.	Australia, Western Pacific	Urban	21	Pregnant women	5	Questionnaire booklet with open questions	C
Cleeton [36]	Suzanne Arms’ 1998 videotape *Giving Birth*	USA, Americas	Sub-Urban	65	Non-pregnant women and male students	Community^a^	Questionnaire with open questions	B

^a^ *Community defined as men and women of reproductive age but not currently pregnant to best of author’s knowledge*

**Table 2:** CERQual summary of findings table

Review finding	Contributing studies	*Assessment confidence in the evidence*	*Explanation of confidence in the evidence assessment*
Theme 1: Mutability of women’s and community’s beliefs about birth: Ambivalence, Empowerment and Fear			
*Women and communities liked learning new information about birth*: The content and design of interventions opened up new ways of thinking about vaginal and caesarean birth for women and communities. Women described how educational interventions informed them about risks and benefits of vaginal birth and caesarean section that were hitherto unknown. Some women were surprised by the actual number of caesareans performed and the risks associated with them. Interventions brought issues of risk to the fore and forced pregnant women in particular to think through more clearly what mattered to them.	[36–47]	High confidence high-income countries and moderate confidence for whole review population	12 studies with minor methodological limitations. Rich data from 7 countries across 3 geographical regions with highest rates of unnecessary caesarean section. No or very minor concerns about coherence.
*Women described pregnancy as an inherent time of uncertainty and transformation of thought about birth method*: While some women described being very sure about their preferred method of delivery prior to or early in the current pregnancy, many of these same women later changed their minds following experience of an intervention or in response to evolving circumstance. Educational interventions played an important role in helping women prepare for any eventually and to reconcile the benefits of their actual birth method when it did not correspond to their preference.	[37, 40–42, 44, 45, 47]	Moderate confidence	7 studies, 4 of which had very minor methodological limitations. Data from Europe and the Americas only. Very minor concerns about coherence.
*Communication of new knowledge and/or support can be empowering:* Learning risks were small, or what labour actually entails, enabled some women to feel more prepared and more confident to labour, especially were information about physiological processes was combined with emotional support. Pregnant or recently delivered women described how they had used information to gain control in the pursuit of informed decision-making; either by using the information to inform more meaningful dialogue with health professionals, or to justify a pre-existing preference for either birth method.	[36–42, 44, 45]	Moderate confidence	9 studies with minor methodological limitations. Sufficiently rich data from 7 countries across 3 geographical regions with highest rates of unnecessary caesarean section. No or very minor concerns about coherence.
*Some information can provoke fear:* Some women and communities found intervention content alarming. Childbirth education video content was described as too gory by a few nulliparous students. Some pregnant women said the use of computer or DVD decision-aids for VBAC increased their anxiety. Use of a decision-aid, combined with follow-up by a midwife helped mediate pregnant women’s concerns about risk in one study, while midwives failing to listen to women’s concerns and forcing them to birth vaginally compounded fears in another.	[36, 38–41, 45, 47]	Moderate confidence	7 studies with minor methodological limitations. Fairly rich data from USA, UK, Taiwan and Norway. Minor concerns about coherence.
Theme 2: Multiplicity of birth information needs: Framing, format and individual management strategies			
*Targeted educational interventions are only one component informing women’s and communities’ views and decision-making about birth method*: Women describe being exposed to a multiplicity of information sources in their pre-, present- and post-pregnancy trajectories. Some women using decision-aids describe them as “a starting point”; a springboard for seeking more information. Learning from the birth stories of family and friends was widespread. Information was also actively sought in the media and from the Internet, while face-to-face interactions with health professionals were viewed as the most important influence on actual birth method.	[36–38, 40–45, 47]	Moderate confidence	10 studies with minor methodological limitations. Fairly rich data 7 countries across 3 geographical regions with highest rates of unnecessary caesarean section. No or very minor concerns about coherence with the other 2 studies not attending to this issue.
*Desire for educational content conveying the physical work of labour and the social and emotional impact of vaginal and caesarean birth for women*. Across settings and education formats, women and communities offered suggestions of what was missing from interventions. They wanted to know more about VBAC and homebirth, what a midwife does, maternity entitlements, the social and emotional impact of caesarean birth, and the “body work” vaginal birth entails. Women also felt vaginal birth could be presented in a more positive way by acknowledging it as an experience. They also wanted information framed in ways women could more easily relate to; for example, many women desired to learn about birth from other women’s experiences; some women wanted information about interventions that was personalised.	[36, 37, 39–47]	High confidence high-income countries and moderate confidence for whole review population	11 studies with minor methodological limitations. Data from 6 countries across 3 geographical regions. Very minor concerns about coherence.
*Women want multiple modes and formats of educational interventions*: Women and communities had wide-ranging views on appropriate language use, figures and tables to communicate information across formats. While many could see the benefits of computer-based interventions, ease of use was problematic for some and pregnant women in particular still desired hard copies of information to revisit and discuss with family members and healthcare professionals. Some concern was expressed about the confidentially of information in on-line decision-aids. Video content was largely welcomed as it facilitated the visualisation of positive, actual birth experiences.	[36–41, 46]	Moderate confidence	7 studies with minor methodological limitations. Data from 3 countries (UK, USA, Taiwan) across three geographical regions. Moderate concerns about adequacy and coherence.
*Women desired emotional support alongside the communication of facts and figures about childbirth:* Women perceived the choice between vaginal birth and caesarean section as huge; with far-reaching consequences for health and wellbeing. Pregnant women in particular described needing emotional support alongside information about the risks and benefits of birth methods. In tandem with interventions women described additional emotional support from husbands, health professionals and doulas.	[36, 38–41, 43, 45]	Moderate confidence	7 studies with minor methodological limitations. Fairly rich data from 5 countries (UK, USA, Australia, Norway and Taiwan). 6 of the 7 studies involved pregnant or post-natal women faced with the gravity of the actual decision-made.
Theme 3: Interactions with health professionals and influence of healthcare system: Support, consistency and autonomy			
*Women welcome health professional’s acknowledgement of previous birth (or life) experience as an important step in decision-making about future birth method*. While previous experiences are important in attitude formation they do not necessarily equate to subsequent preference for delivery method. Across study settings many women and communities valued vaginal birth as natural and a meaningful life experience for women, with fears associated with labour and vaginal birth (pain, uterine rupture) not insurmountable. Few women categorically preferred caesarean section. Some women who had previous experience of caesarean section were particularly keen to avoid it.	[36–38, 40–45, 47]	Moderate confidence	10 studies with minor methodological limitations. Data from 7 countries across 3 geographical regions with richest data from European settings. Minor concerns about coherence.
*Intervention content was most useful when it complemented clinical care, was consistent with advice from health professionals and provided a basis for more informed, meaningful dialogue between women and care providers*: Some women and communities experiences of interventions suggest they raised more questions than they answered and created a need for additional dialogue with health professionals to discuss issues raised, fears evoked, and revisit birth plans. While some pregnant women described themselves as “desperate” for such conversations, other women were dissatisfied when their expectations went unmet because conversations were too brief, their views were not listened too, the health professional was unknown to them, and/or gave inconsistent advice.	[36, 37, 40, 41, 43, 45–47]	Moderate confidence	8 studies with minor methodological limitations. Data from 4 countries across 3 geographical regions. No or very minor concerns about coherence.
*Women’s attitude towards involvement in decision-making*: Some women have a strong desire to be involved and to exert control in the decision-making process; others are less certain of their role and value some involvement; while others still are reluctant for any involvement and want qualified health professionals to make the decision for them. The success of any intervention to reduce unnecessary caesarean section is dependent upon pregnant women being open to a role in decision-making and some degree of uncertainty surrounding preference for caesarean section.	[37, 38, 40, 41, 43–47]	Moderate confidence	9 studies with minor methodological limitations. Data from 7 countries across 3 geographical regions. Minor concerns about coherence.
*Women are aware of how the organisation of care and information impacts the actual choices available to them*: Some women and communities believed intervention content favoured health professionals’ hidden agendas to promote whichever method of birth was favoured by them or the hospital or health system in which they work. In two geographical regions pregnant women used metaphors of conflict in the pursuit of their choice of birth method. Other women questioned the exclusion of information about homebirth, excessively high caesarean section rates, and why doctors aren’t publically accountable for the number of caesarean sections performed if they are “cutting on women” unnecessarily.	[36–41, 43–45, 47]	Moderate confidence	10 studies with minor methodological limitations. Data from 7 countries across 3 geographical regions. Moderate concerns about coherence.

### Theme 1: Mutability of women’s and community’s beliefs about birth: Ambivalence, empowerment and fear

This theme encapsulates how beliefs about birth vary across populations and over time. Individual women’s views about vaginal and caesarean birth are neither stable nor mutually exclusive, as information is continuously and concurrently communicated to them pre-, during, and post-pregnancy.

#### Women and communities like learning new information about birth

Participants talked about how interventions had opened up new ways of thinking about birth for them, irrespective of parity, [36–48] with some surprised by the actual number of CSs performed and the risks associated with them. This view was typified by a nulliparous woman in the USA: “*I didn’t realize there were so many C-sections. I kind of always thought that was like the last option*” ([48]: p. 128). Intervention content also brought issues of risk to the fore as illustrated by this participant in the DiAMOND Trial [50] who initially wanted a VBAC, but when the Decision-Aid (DA) proposed an elective CS, changed her mind: “*It [DA] educated me in risks that I didn’t know about… it’s nice to have been able to do this and learn the things*” ([41]: p. 900).

#### Pregnancy as a time of uncertainty and transformation of thought about birth

Participants in seven studies [37, 40–42, 44, 45, 48] reported a preference for a particular birth method prior to, or early in pregnancy, then later changed their minds. Three of these studies exclusively involved women with a previous CS [37, 40, 41]. Two studies reported women who were mildly in favour of a VBAC or who were unsure, changed their preference to an elective CS after the intervention [37, 41]. In one study of non-pregnant women in the community, the change reported was from a preference for CS, to a preference for vaginal birth [42]. Some women talked about the nature of pregnancy as inherently uncertain [40, 41, 44]. Educational interventions had the additional benefit of helping prepare for any eventuality, and to reconcile the benefits of their actual birth method if it did not correspond to their prior preference [41, 44].

#### Communication of new knowledge, education and support can be empowering

Some women reported feeling more prepared to labour after intervention exposure, especially where information about physiological processes was combined with support from a midwife or doula [36–42, 44, 45]. Educational content facilitated shifts in confidence, as expressed by a Taiwanese multiparous woman, who “*felt a lot of pain and lost confidence when giving birth last time. I felt very different this time. Because I had taken this course I felt very confident when I was giving birth*” ([38]:p5). Similarly, a nulliparous college student in the US commented *“this film… turns it [pain] into a form of “power” for women”* ([36]:p197). Pregnant, and new mothers also reported how they had used information to gain control; either by using educational content to inform more meaningful dialogue with health professionals, or to justify a pre-existing preference for CS or vaginal birth [40, 41].

#### Educational intervention content as anxiety provoking

Childbirth education video content was described as “too graphic” by a few nulliparous students, [36] while some pregnant women described how the use of computer or DVD decision-aid increased their anxiety by communicating “scary information” [40, 41]. Midwifery support helped mediate some pregnant women’s concerns, [40, 45] but when midwives and health professionals were perceived as not listening to women’s concerns it compounded their fear [45].

This theme highlights the potential of educational interventions to increase anxiety, but also to have positive effects including enhanced knowledge, transformation of beliefs, and confidence to labour, which could reduce CS rates. However, the underlying mechanisms of mutability meant the same interventions were also used in support of elective CS. Reasons for these inconsistencies in effect are explored in theme 2.

### Theme 2: Multiplicity of birth information needs: Framing, format and individual management strategies

This theme encapsulates how recipients of educational interventions differ in terms of levels of health literacy (i.e. familiarity with medical terms), demand for information, and uptake. It also demonstrates how the content is neither delivered, nor received, in a vacuum.

#### Educational interventions are only one component informing decision-making about birth method

Women described being exposed to a multiplicity of sources of information [36–38, 40–45, 47]. Women using decision-aids described them as “*a starting point”*; a springboard for seeking more information ([41]:p899). Learning from the birth stories of family and friends was widespread, as was seeking information from the Internet. Face-to-face interactions with health professionals were viewed as the most important influence on actual birth method. Discussions occurring at 36 week antenatal appointments were reported in the UK as limiting the effects of a decision-support intervention delivered earlier in pregnancy ([40]:p121).

#### Desire for educational content conveying the physical work of labour and the social and emotional impact of vaginal and caesarean birth

Within and between studies participants identified missing components, including what a midwife does, maternity entitlements, the social and emotional impact of CS, and the physical labour of vaginal birth [36, 37, 39–41, 43, 45–47]. Some participants thought information about vaginal birth should be communicated less as “a medical phenomenon” and more as a personal, spiritual, and emotional experience ([36]:p196). Across interventions, participants wanted information framed in ways they could more easily relate to, particularly watching or reading “*responses from* [real] *people*” ([46]:p:394). Interventions with components where obstetric histories and personal characteristics (age, height) could be entered, were welcomed by some, but not all women. If the outcome of the exercise didn’t fit with women’s prior decisions, they were less likely to find it helpful [41, 46].

#### Women want multiple modes and formats of educational interventions

Different women reported different levels of literacy, comprehension or requisite skills and access to electronic resources. While many women reported benefits of computer-based interventions, [38, 39, 41, 46] ease of use was problematic for some [39, 41] as were confidentiality concerns, [46] and an unmet need for hard copies to reflect upon, revisit and share information during discussions with family, friends and health professionals [39, 46]. Varied views on the appropriateness of language, figures, tables, and quizzes to communicate information across formats were reported [36, 38–40, 46]. Some women talked about wanting “*not medical terms, kind of straight talking terms and easy facts*”, found terms like “*perinatal unclear”*, or “*out of every thousand women like you patronising*” ([39]:p167). Video content was mainly welcomed because it facilitated the visualisation of positive, actual birth experiences, and was easily understood [36, 38, 40].

#### Desire for emotional support alongside the communication of facts and figures about birth

Women talked about the decision between vaginal or caesarean birth in the context of their lives [36, 38–43, 45]. Pregnant women in particular described needing emotional support alongside information about the risks and benefits of birth methods. Women with a previous CS specifically needed someone to listen to their previous birth experience, to help them understand what had happened to them and why, to help them go forward in their current pregnancy [40, 43, 45] with midwifery emotional support acknowledged by some as a “*turning point*” from preferring another CS to going ahead with a planned vaginal birth [40, 45]. Women valued emotional support from their partners, health professionals and doulas [38–41, 43, 45].

This theme demonstrates the multiplicity of women’s birth education needs, the role of individual agency in seeking and managing information, and the importance of how information is communicated. The role of health professional support is explored further in theme 3, alongside the need for consistency between the information imparted in educational interventions and in clinical care, and women’s perceptions of who has autonomy over the choice of actual birth method.

### Theme 3: Interactions with health professionals and influence of healthcare system: Support, consistency and autonomy

This final theme reports how shifts in views about birth method are partly shaped by routine interactions with health professionals and health system factors.

#### Women welcome health professional’s acknowledgement of previous birth (and life) experiences

Women wanted health professionals to acknowledge their prior beliefs and experiences, especially previous traumatic birth experiences, *“for the massive thing that it is”* to them ([40]:p.120). Previous experiences were important in attitude formation but they did not necessarily equate to subsequent preference for delivery method [36–38, 40–45, 47]. For example, a Norwegian woman (expecting her third child) who requested a CS came to think vaginal birth was possible “*on my own terms*” ([45]:p271) as a result of the intervention, because she felt her midwife now “knew how she felt” ([45]:p271) and was able to organise care in such a way as to overcome her specific fears. Across study settings many women and communities valued vaginal birth as natural and a meaningful life experience [36–38, 42–44, 47]. While they expressed fears associated with labour and vaginal birth (pain, uterine rupture), they felt these fears could be allayed with appropriate information and support. Few women categorically preferred CS and some with a history of CS were keen to avoid it [37–41, 43].

#### Intervention content is most useful when it complements clinical care, is consistent with advice from health professionals and provides a basis for more informed, meaningful dialogue between women and care providers

As reported in Theme 1, interventions could generate more questions than they answered, creating the need for more dialogue with health professionals [36, 37, 40, 41, 43, 45–47]. Multiparous women discussed the tension between receiving fewer opportunities for consultation with health professionals (compared to their first pregnancy), yet feeling there was more to discuss [40, 41]. Some pregnant women described themselves as *“desperate”* for such conversations, with they and others dissatisfied when their expectations went unmet [36, 37, 40, 41, 45–47].

#### Women’s attitudes towards involvement in decision-making

Some women were highly motivated to be involved in decision-making about birth method, [37, 40, 41, 43, 45] others uncertain of their role or wanted a healthcare professional to make the decision for them [37, 38, 40, 41, 43–47]. Reasons for reluctance included respect for professional knowledge, in contrast to their own, confusion about their right to choose, and readiness to simply see how pregnancy and birth goes.

#### Women are aware of how the organisation of care and information can impact the actual choices available to them

Some women and communities were suspicious that intervention content favoured health professionals, hospital or health systems hidden agendas [36–41, 43–45, 47]. Women used metaphors of conflict to describe their perception of the need to engage with health services “*armed with information*” ([43]:p168) in pursuit of real choice. They anticipated having a “*fight on your hands*,” ([40]:p121) and “*dialogue [is] the only weapon*” ([44]:p396). Women and men also questioned why doctors are not more publically accountable for “*cutting on women*” ([48]:p132).

This theme shows recipients of educational interventions understand how health professional preferences and health systems can influence actual birth method. In the final interpretive synthesis stage of the analysis findings were combined to represent our interpretation, through a line of argument.

### Line of argument synthesis

Educational interventions targeted at women are one of multiple factors that influence highly emotive, complex, and fluid decision-making processes during pregnancy and childbirth. In this dynamic, multi-layer context, focused, linear interventions may have limited effects. Prior birth or life experiences, appeared to be important in guiding interpretation of educational materials, including their use to reinforce an existing preference for birth method. They can be empowering and instigate confidence. They also seemed helpful in enabling women to confront the issues where there was ambivalence for birth method. Pregnant and non-pregnant recipients of educational interventions suggest that accurate content is necessary, but not sufficient. Recipients also needed to trust in the information being communicated which may be challenging in the context of certain health systems and alleged hidden agendas. For maternity service users, meaningful interactions with wider social networks, particularly health professionals, had the potential to frame the educational interventions, and to transform expectations and experiences of actual birth method.

## Discussion

### Main findings

An important facilitator of interventions was the widespread appeal of learning about birth. However, this could lead to anxiety as well as empowerment. The acceptability of educational intervention formats and content varied, but a common thread was the importance of communicating information that matters to women. Prior birth experiences, routine interactions (with social networks, health professionals) and continuing information communication (during antenatal appointments, from books or the Internet) of birth information appeared especially important in framing perceptions of intervention value. The potential for the transformation of beliefs about birth, pregnant women’s desire for emotional support, and need for dialogue with health professionals acted as facilitators or barriers to positive educational intervention effects. Important barriers to intervention success were inconsistent information content (especially between intervention and routine care) and mistrust in the healthcare system, based on the belief that it would not deliver birth method of choice if this choice did not fit system norms.

### Strengths and limitations

The use of GRADE-CERQual assessment of confidence in findings in this synthesis is a strength [37, 51]. The transferability of the findings across resource settings is limited because eleven of the twelve included studies were from high-income countries where greater emphasis has been placed on women’s involvement in decision-making during pregnancy and childbirth. Only one included study was from a middle-income country, Brazil, where rates of CS are particularly high. Furthermore Brazil is considered an upper-middle income country because of the progressive rise of many of its indicators. No studies were from low-income settings, and there were no non-English language publications. The paucity of included studies is an important limitation.

### Interpretation

The present synthesis adds to the emergent evidence-base on reducing unnecessary CS [2–7, 9, 21]. The updated Cochrane review of non-clinical indications to reduce unnecessary CS [9], found only three of the twelve interventions targeted at women or communities had a desirable effect. The qualitative evidence presented in this synthesis suggests women may value emotional experience of care as much as actual birth method per se as an outcome. Women’s right to choose their birth method is an important topic in the general CS literature. However, this was not a prominent theme in the current study. Instead women were looking for meaningful and continued interactions with health professionals who could be trusted to provide advice consistent with the educational information received. The review demonstrates that the wider health system (including health professionals) is an important (positive and negative) determinant of women’s views and decision-making about birth. This finding resonates with wider literature reporting women’s and health professional’s views of the reasons behind CS rates [52–54].

The findings also have resonance with existing evidence that women perceive decision-making about birth method as problematic, and that variations exist between what women want to know and what health professionals believe that they should know [55, 56]. Previous studies have suggested that women particularly note a lack information about the benefits of vaginal birth. The absence of evidence on an optimal education format is identified as particularly concerning in quantitative systematic reviews [9]. One systematic review that includes quantitative and qualitative studies suggests the potential of different tools to improve knowledge, and reduce anxiety or decisional-conflict when used at key pregnancy decision points, and in specific circumstances [57]. Women with a previous caesarean (Robson Group 5) could benefit from the more intensive decisional support process these tools facilitate when used as an adjunct to clinical counselling. They can offer women (and clinicians) the opportunity to address their anxieties and the time to consider choice of birth method in partnership.

There has been considerable technological advance during the timespan of the included studies. This synthesis shows how women seek information from multiple sources. The media and communications industry has become a major distributor of information about birth since 2001, with mounting professional concerns about the accuracy and completeness of information [58–60]. A recent meta-synthesis of informal information sources and women’s decision-making about birth also suggests the need for a more central role for healthcare providers in the curation and unification of trustworthy information [61]. We identified no eligible studies of celebrity endorsement of birth method, or public dissemination of rates of birth type, but these may prove to be powerful educational tools in the future [16, 62, 63].

Formative qualitative research in local contexts could help improve the design of educational interventions [39, 46, 54] and help build trust, between women, communities and health professionals. It is unclear if the formats discussed here would be accessible in middle and low-income settings, or the availability of providers with time to explain information, and the appropriateness of some content. Future research should focus on evaluating multi-faceted strategies simultaneously targeting women, health professionals, and system change [64–70].

## Conclusion

Women’s values and preferences for birth, and for information format and content, vary across populations, and in individual women over time. Unpredictable shifts in views are partly shaped by interactions with health professionals, and by health system factors, as well as specific educational materials. Educational interventions that do not take account of these dynamic factors may have limited effects on the rising CS rate. Tackling unnecessary CS requires educational information with a consistent message, but tailored for the specific needs, values and beliefs of women and communities, alongside emotional support, delivered how and when women need it, in dialogue with health professionals, and reinforced by the health systems encountered by women throughout their pregnancies.

## Additional files

Additional file 1: **Appendix S0.** PRISMA checklist. (DOCX 32 kb)

Additional file 2: **Appendix S1.** Search strategy. (DOCX 32 kb)

Additional file 3: **Figure S1.** Robson 10 Group Classification. (JPG 3213 kb)

Additional file 4: **Box S1.** Summary of qualitative synthesis process. (DOCX 26 kb)

Additional file 5: **Table S1.** Themes, emergent themes, initial concepts and supporting quotes. (DOCX 35 kb)

Additional file 6: **Table S2.** CERQual Summary of evidence profile table. (DOCX 33 kb)

## Abbreviations

CERQual: Confidence in the Evidence from Reviews of Qualitative research
CS: Caesarean section
