# Supplementary material for: Women’s and communities’ views of targeted educational interventions to reduce unnecessary caesarean section: a qualitative evidence synthesis
Source: Reprod Health. 2018 Jul 24;15:130. doi: 10.1186/s12978-018-0570-z (PMC6057083; doi:10.1186/s12978-018-0570-z)
Supplement: Supplementary file 2 — Appendix S1. Search strategy. (DOCX 32 kb) [file 12978_2018_570_MOESM2_ESM.docx]

**Additional Information (Appendix 1: Search strategy CINAHL Complete (EBSCO*host)*)**

| # | Query | Limiters/Expanders | Last run via | Results |
| --- | --- | --- | --- | --- |
| S1 | (MH "Women+") OR "woman" | Search modes - Boolean/Phrase | Interface - EBSCOhost Research Databases  Search Screen - Advanced Search  Database - CINAHL Complete | 48,423 |
| S2 | (MH "Expectant Mothers") OR (MH "Expectant Parents+") OR (MH "Expectant Fathers") OR (MH "Mothers+") | Search modes - Boolean/Phrase | Interface - EBSCOhost Research Databases  Search Screen - Advanced Search  Database - CINAHL Complete | 26,493 |
| S3 | (MH "Maternal Attitudes") OR "maternal" | Search modes - Boolean/Phrase | Interface - EBSCOhost Research Databases  Search Screen - Advanced Search  Database - CINAHL Complete | 61,963 |
| S4 | (MH "Fathers+") OR "father" | Search modes - Boolean/Phrase | Interface - EBSCOhost Research Databases  Search Screen - Advanced Search  Database - CINAHL Complete | 8,739 |
| S5 | (MH "Communities+") | Search modes - Boolean/Phrase | Interface - EBSCOhost Research Databases  Search Screen - Advanced Search  Database - CINAHL Complete | 34,885 |
| S6 | (MH "Public Policy+") OR (MH "Public Opinion") OR (MH "Public Relations+") OR "public" | Search modes - Boolean/Phrase | Interface - EBSCOhost Research Databases  Search Screen - Advanced Search  Database - CINAHL Complete | 313,760 |
| S7 | S1 OR S2 OR S3 OR S4 OR S5 OR S6 | Search modes - Boolean/Phrase | Interface - EBSCOhost Research Databases  Search Screen - Advanced Search  Database - CINAHL Complete | 460,465 |
| S8 | (MH "Personnel, Health Facility+"# OR #MH "Attitude of Health Personnel+"# OR #MH "Medical Staff+"# OR #MH "Staff Nurses"# OR #MH "Staff Development+"# OR "staff" | Search modes - Boolean/Phrase | Interface - EBSCOhost Research Databases  Search Screen - Advanced Search  Database - CINAHL Complete | 171,947 |
| S9 | (MH "Organizational Culture+") OR "organization" | Search modes - Boolean/Phrase | Interface - EBSCOhost Research Databases  Search Screen - Advanced Search  Database - CINAHL Complete | 59,785 |
| S10 | (MH "Personnel, Health Facility+"# OR #MH "Hospital Units+") OR "facility" | Search modes - Boolean/Phrase | Interface - EBSCOhost Research Databases  Search Screen - Advanced Search  Database - CINAHL Complete | 134,476 |
| S11 | (MH "Midwife Attitudes") OR (MH "Nurse Midwives") OR (MH "Midwives") OR (MH "Midwifery Service") OR (MH "Education, Nurse Midwifery") OR "midwife" | Search modes - Boolean/Phrase | Interface - EBSCOhost Research Databases  Search Screen - Advanced Search  Database - CINAHL Complete | 15,040 |
| S12 | (MH "Physician Attitudes") OR "Physician" | Search modes - Boolean/Phrase | Interface - EBSCOhost Research Databases  Search Screen - Advanced Search  Database - CINAHL Complete | 83,592 |
| S13 | (MM "Health Systems Agencies") | Search modes - Boolean/Phrase | Interface - EBSCOhost Research Databases  Search Screen - Advanced Search  Database - CINAHL Complete | 203 |
| S14 | (MH "Multidisciplinary Care Team+") OR "health care provider" | Search modes - Boolean/Phrase | Interface - EBSCOhost Research Databases  Search Screen - Advanced Search  Database - CINAHL Complete | 34,717 |
| S15 | S8 OR S9 OR S10 OR S11 OR S12 OR S13 OR S14 | Search modes - Boolean/Phrase | Interface - EBSCOhost Research Databases  Search Screen - Advanced Search  Database - CINAHL Complete | 414,412 |
| S16 | S7 OR S15 | Search modes - Boolean/Phrase | Interface - EBSCOhost Research Databases  Search Screen - Advanced Search  Database - CINAHL Complete | 815,501 |
| S17 | (MH "Early Intervention+") OR (MH "Intervention Trials") OR (MH "Nursing Interventions") OR (MH "Experimental Studies+") OR "Intervention" | Search modes - Boolean/Phrase | Interface - EBSCOhost Research Databases  Search Screen - Advanced Search  Database - CINAHL Complete | 378,666 |
| S18 | (MH "Program Evaluation") OR (MH "Summative Evaluation Research") OR (MH "Formative Evaluation Research") OR (MH "Evaluation Research+") OR "programme evaluation" | Search modes - Boolean/Phrase | Interface - EBSCOhost Research Databases  Search Screen - Advanced Search  Database - CINAHL Complete | 72,501 |
| S19 | (MH "Quality Improvement+") OR (MH "Clinical Documentation Improvement") OR (MH "Evaluation and Quality Improvement Program") OR (MH "Change Management") OR "improvement" | Search modes - Boolean/Phrase | Interface - EBSCOhost Research Databases  Search Screen - Advanced Search  Database - CINAHL Complete | 121,753 |
| S20 | (MH "Organizational Change") | Search modes - Boolean/Phrase | Interface - EBSCOhost Research Databases  Search Screen - Advanced Search  Database - CINAHL Complete | 9,832 |
| S21 | (MH "Patient Education+") | Search modes - Boolean/Phrase | Interface - EBSCOhost Research Databases  Search Screen - Advanced Search  Database - CINAHL Complete | 64,052 |
| S22 | (MH "Decision Support Techniques+") OR (MH "Decision Support Systems, Clinical") OR (MH "Decision Support Systems, Management") OR (MH "Decision Making, Organizational") OR "decision aids" | Search modes - Boolean/Phrase | Interface - EBSCOhost Research Databases  Search Screen - Advanced Search  Database - CINAHL Complete | 12,332 |
| S23 | (MH "Education, Nursing, Continuing") OR (MH "Education, Medical, Continuing") OR (MH "Education, Continuing+") OR "continuing professional education" | Search modes - Boolean/Phrase | Interface - EBSCOhost Research Databases  Search Screen - Advanced Search  Database - CINAHL Complete | 27,501 |
| S24 | (MH "Clinical Competence+") OR (MH "Practice Patterns") OR (MH "Clinical Exemplars") OR (MH "Teaching Materials, Clinical") OR (MH "Clinical Assessment Tools+") OR "clinical audit" | Search modes - Boolean/Phrase | Interface - EBSCOhost Research Databases  Search Screen - Advanced Search  Database - CINAHL Complete | 210,091 |
| S25 | (MH "Practice Guidelines") OR (MH "Guideline Adherence") OR (MH "Public Policy") OR (MH "Policy Making") OR "guidelines" | Search modes - Boolean/Phrase | Interface - EBSCOhost Research Databases  Search Screen - Advanced Search  Database - CINAHL Complete | 138,839 |
| S26 | (MH "Harm Reduction") OR "reduce" | Search modes - Boolean/Phrase | Interface - EBSCOhost Research Databases  Search Screen - Advanced Search  Database - CINAHL Complete | 94,257 |
| S27 | (MH "Public Opinion") OR (MH "Referral and Consultation+") OR "routine second opinion" | Search modes - Boolean/Phrase | Interface - EBSCOhost Research Databases  Search Screen - Advanced Search  Database - CINAHL Complete | 33,876 |
| S28 | barriers or obstacles or challenges | Search modes - Boolean/Phrase | Interface - EBSCOhost Research Databases  Search Screen - Advanced Search  Database - CINAHL Complete | 150,472 |
| S29 | facilitators or motivators | Search modes - Boolean/Phrase | Interface - EBSCOhost Research Databases  Search Screen - Advanced Search  Database - CINAHL Complete | 7,344 |
| S30 | S17 OR S18 OR S19 OR S20 OR S21 OR S22 OR S23 OR S24 OR S25 OR S26 OR S27 OR S28 OR S29 | Search modes - Boolean/Phrase | Interface - EBSCOhost Research Databases  Search Screen - Advanced Search  Database - CINAHL Complete | 1,069,163 |
| S31 | Cesarean | Search modes - Boolean/Phrase | Interface - EBSCOhost Research Databases  Search Screen - Advanced Search  Database - CINAHL Complete | 16,553 |
| S32 | (MH "Cesarean Section+"# OR #MH "Cesarean Section, Repeat"# OR #MH "Vaginal Birth After Cesarean"# OR #MH "Cesarean Section, Elective"# OR "cesarean" | Search modes - Boolean/Phrase | Interface - EBSCOhost Research Databases  Search Screen - Advanced Search  Database - CINAHL Complete | 16,532 |
| S33 | (MH "Childbirth+") OR "childbirth" OR (MH "Childbirth Educators") OR (MH "Childbirth Education") OR (MH "Home Childbirth") | Search modes - Boolean/Phrase | Interface - EBSCOhost Research Databases  Search Screen - Advanced Search  Database - CINAHL Complete | 26,326 |
| S34 | S31 OR S32 OR S33 | Search modes - Boolean/Phrase | Interface - EBSCOhost Research Databases  Search Screen - Advanced Search  Database - CINAHL Complete | 39,207 |
| S35 | qualitative research | Search modes - Boolean/Phrase | Interface - EBSCOhost Research Databases  Search Screen - Advanced Search  Database - CINAHL Complete | 10,083 |
| S36 | (MH "Structured Interview") OR (MH "Interviews+") OR "interviews" OR (MH "Unstructured Interview") OR (MH "Semi-Structured Interview") | Search modes - Boolean/Phrase | Interface - EBSCOhost Research Databases  Search Screen - Advanced Search  Database - CINAHL Complete | 184,622 |
| S37 | (MH "Attitude") OR (MH "Behavior+") OR (MH "Attitude of Health Personnel") OR (MH "Family Attitudes") OR (MH "Social Values+") | Search modes - Boolean/Phrase | Interface - EBSCOhost Research Databases  Search Screen - Advanced Search  Database - CINAHL Complete | 701,738 |
| S38 | qualitative or case study or interview or observation or focus group or ethnograph or case study | Search modes - Boolean/Phrase | Interface - EBSCOhost Research Databases  Search Screen - Advanced Search  Database - CINAHL Complete | 400,983 |
| S39 | (MH "Qualitative Studies+") OR "qualitative" | Search modes - Boolean/Phrase | Interface - EBSCOhost Research Databases  Search Screen - Advanced Search  Database - CINAHL Complete | 126,780 |
| S40 | view* OR want* OR cho* OR prefer* OR feel* OR thought* OR like OR accept* OR dislike OR wish OR hope or fear | Search modes - Boolean/Phrase | Interface - EBSCOhost Research Databases  Search Screen - Advanced Search  Database - CINAHL Complete | 450,000 |
| S41 | S35 OR S36 OR S37 OR S38 OR S39 OR S40 | Search modes - Boolean/Phrase | Interface - EBSCOhost Research Databases  Search Screen - Advanced Search  Database - CINAHL Complete | 1,261,828 |
| S42 | S34 AND S41 | Search modes - Boolean/Phrase | Interface - EBSCOhost Research Databases  Search Screen - Advanced Search  Database - CINAHL Complete | 11,721 |
| S43 | S16 AND S30 AND S42 | Limiters - Published Date: 19850101-20171231  Search modes - Boolean/Phrase | Interface - EBSCOhost Research Databases  Search Screen - Advanced Search  Database - CINAHL Complete | 2,225 |
| S89 | S17 OR S21 | Search modes - Boolean/Phrase | Interface - EBSCOhost Research Databases  Search Screen - Advanced Search  Database - CINAHL Complete | 433,186 |
| S90 | S7 AND S34 AND S41 AND S89 | Search modes - Boolean/Phrase | Interface - EBSCOhost Research Databases  Search Screen - Advanced Search  Database - CINAHL Complete | 873 |
| S91 | S7 AND S31 AND S41 AND S89 | Search modes - Boolean/Phrase | Interface - EBSCOhost Research Databases  Search Screen - Advanced Search  Database - CINAHL Complete | 314 |
