# Supplementary figures and images for: Women’s and communities’ views of targeted educational interventions to reduce unnecessary caesarean section: a qualitative evidence synthesis
Source: Reprod Health. 2018 Jul 24;15:130. doi: 10.1186/s12978-018-0570-z (PMC6057083; doi:10.1186/s12978-018-0570-z)

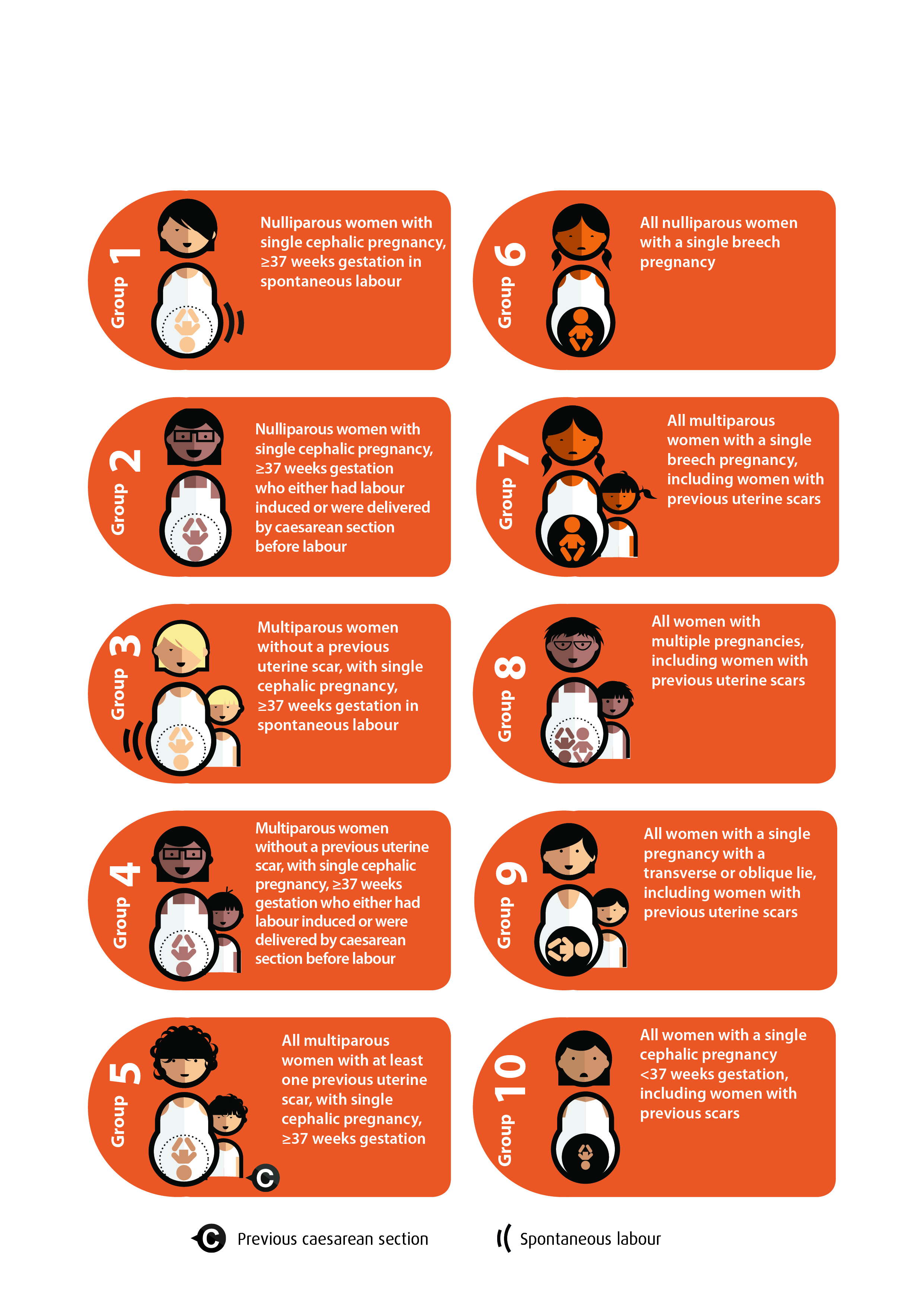

Supplement: Supplementary file 3 — Figure S1. Robson 10 Group Classification. (JPG 3213 kb) [file 12978_2018_570_MOESM3_ESM.jpg]
