# Supplementary material for: Women’s and communities’ views of targeted educational interventions to reduce unnecessary caesarean section: a qualitative evidence synthesis
Source: Reprod Health. 2018 Jul 24;15:130. doi: 10.1186/s12978-018-0570-z (PMC6057083; doi:10.1186/s12978-018-0570-z)
Supplement: Supplementary file 4 — Box S1. Summary of qualitative synthesis process. (DOCX 26 kb) [file 12978_2018_570_MOESM4_ESM.docx]

**Additional Information 3**

**Box 1: Summary of qualitative synthesis process**

**1.Familarisation and quality assessment**

This stage was done at the level of individual studies. In order to be included, each study had to have used qualitative methods for both data collection and for data analysis. This criterion constituted a basic quality threshold. In addition, formal methodological quality assessment of studies was undertaken by applying a quality appraisal framework to each study. This task was initially undertaken by the lead author(CK), then a second review author(SD) checked for discrepancies. The criteria from Walsh [66] and the A-D grading of Downe [67] was used to assess study scope and purpose, design, sampling strategy, analysis, interpretation, researcher reflexivity, ethical dimensions, relevance, and transferability. While no studies meeting the inclusion criteria were excluded on study quality, these assessments were used when judging the relative contributions of each study to the development of explanations and relationships between the studies. This meant the synthesis is “weighted” towards the findings of the better quality studies [68]. Following the principles of meta-ethnography [18] data extraction and analyses were undertaken simultaneously (stages 2-4). Meta-ethnography uses an approach based on the constant comparative technique, in which the analysis is built up study by study, using the principles of confirmation ('reciprocal analysis') and dis-confirmation ('refutational analysis').

***2. Data extraction*** Characteristics of included studies were recorded using a form designed specifically for this review, adapted from those used in earlier mixed-methods reviews [69-70]. Starting with the earliest published paper [36] for each included study, relevant verbatim text (participant quotes), and author interpretation (themes, theories and metaphors) were extracted.

***3. Coding*** Extracted data was coded for initial concepts by CK. Codes were developed using extracted data from the first paper and then comparing it with the findings from another until all extracted data from all included studies was coded. Code labels were then discussed, refined and agreed by consensus with SD.

***4. Interpretative synthesis*** Initial codes were grouped into emergent themes, first by reciprocal analysis (confirmatory of similarities in data across studies) and then by refutational analysis (integration of disconfirmatory data into emergent themes). Emergent themes were then summarised for CERQUal assessment in Stage 5. Before this was undertaken, CK and SD synthesised the emergent themes into final themes and the resultant thematic structure into a ‘line of argument’ synthesis.

***5. GRADE Confidence in the Evidence from Reviews of Qualitative research*** GRADE-CERQual [35,51] is an approach to assess the confidence in qualitative evidence synthesis findings. Assessment is at the level of review findings (also known as emergent themes). CERQual assessment comprises of four domains: 1)Methodological limitations - The extent to which there are problems in the design or conduct of the primary studies that contributed evidence to a review finding; 2)Relevance - The extent to which the body of evidence from the primary studies supporting a review finding is applicable to the context (perspective or population, phenomenon of interest, setting) specified in the review question; 3)Coherence - The extent to which the review finding is well grounded in data from the contributing primary studies and provides a convincing explanation for the patterns found in these data; 4)Adequacy – The assessment of the degree of richness and quantity of data supporting a review finding.
