# Supplementary material for: Women’s and communities’ views of targeted educational interventions to reduce unnecessary caesarean section: a qualitative evidence synthesis
Source: Reprod Health. 2018 Jul 24;15:130. doi: 10.1186/s12978-018-0570-z (PMC6057083; doi:10.1186/s12978-018-0570-z)
Supplement: Supplementary file 5 — Table S1. Themes, emergent themes, initial concepts and supporting quotes. (DOCX 35 kb) [file 12978_2018_570_MOESM5_ESM.docx]

**Additional information 4 - Table A1: Final themes, emergent themes, and initial concepts (with supporting quotes)**

| **Initial concepts** | **Emergent Themes/Summary of Review Findings** | **Papers** | **Illustrative quotes** | **Final Themes** |
| --- | --- | --- | --- | --- |
| Learned something new | Women and communities like learning new information about birth | 36,37,38,39,  40,41,42,43,  44,45,46,47 | "I thought it was really good actually, really helpful” (Emmett 2007:167)  “It was good to make me think about it” (Frost 2009:899).  “So I decided not to take C/S even though my last C/S is OK. Now I knew giving natural birth is better.” (Wang 2006:6)  “I never knew that giving regular birth protects the baby some type of way versus you having a C-section.” (McCants 2016:128)  “I learned that there is a difference between a “normal” birth and a “typical” birth.” (Cleeton 2001:196)  "I learned that the risks of caesarean section are higher than vaginal delivery. I thought it was the other way around." (Milne 2009:6) | Mutability of women’s and communities beliefs about birth |
| Made women confront issues in certain way |  |  |  |  |
| Surprise at what learnt |  |  |  |  |
| Same decision-aid used in different ways | Women describe pregnancy as a time of uncertainty, when beliefs and views about birth can change as pregnancy develops | 37,40,41,42,44,45,47 | “I want to try normal delivery, but I want to know everything about the development of labor, to understand the reasons that lead to a C-section”(Basso 2010:393.)  Most women found themselves dealing with uncertainty and difficulty in making the decision (Farnworth, 2008:120)  Women interpreted this mismatch (between DA and actual delivery method) as symbolic of the inherent uncertainty associated with planning a delivery (Frost 2009:901-2). |  |
| Uncertainty inherent in childbirth |  |  |  |  |
| Transformation (preference, actual birth method) |  |  |  |  |
| New knowledge empowered women to believe vaginal birth desirable | The new knowledge and support communicated in educational interventions can be empowering for women | 36,37,38,39,40,41,42,44, 45 | I felt confident about my child birth because I knew the next step of child birth” (Wang 2006:6)  Information combined with support gave women confidence in their decision, and ultimately, the power to own and justify the decision they had made (Farnworth 2008:120).  “The information that I had got from the programme, I felt empowered by it, if you can put it like that” (Frost 2009:902) |  |
| New knowledge empowered women to pursue choice of birth method (VB or CS) |  |  |  |  |
| Intervention made individuals more fearful of a particular birth method | Some women and communities experience educational intervention content as anxiety provoking | 36,38,39,40,41,45,47 | “I wasn’t particularly happy with [Decision analysis] at all. I thought a lot of the things, was just a lot of scary information” (Frost 2009:900)  “You could get yourself quite wound up about it all" (Emmett 2007:168)  “I cried a lot, was completely torn apart, and could not say anything” (Ramvi2011:271)  ‘‘Too graphic for me!’’ ”Students should definitely be warned before they watch it.’’ (Cleeton 2001:196) |  |
| Learning about birth perceived as too gory |  |  |  |  |
| Other sources of information from family, friends, health professionals, Internet | Educational interventions are only one component informing women’s and communities’ views and decision-making about birth | 36,37,38,40,41,42,43,44,45,47 | “If we evaluate birth as it currently occurs, we realize that we need to seek information so that our will is respected” (Basso 2010:395)  "I was left thinking: right, I want more information now, I want to know more. So then I went onto the internet…And also, in the book that I bought for my first pregnancy” (Frost 2009:899)  ‘‘What research is there that shows the effects of an epidural on a baby?’’ (Cleeton 2001) | Multiplicity of women’s and communities birth information needs |
| Educational intervention beginning, not end of information sought |  |  |  |  |
| Need for more and different information (i.e. home birth, spiritual aspects of birth) | Women want educational booklets, workshops and decision-aids conveying the physical work of labour and the social and emotional impact of vaginal birth and caesarean section | 36,37,39,40,41,42,43,44,45,46,47 | “What I tend to think about are more practical things like, you know, being able to pick up the baby after, after an operation or getting an infection” (Frost 2009:900)  “I like it better when I can put my own statistics in and get my own results” (Shorten, 2015:394)  “So, getting prepared through body movements, before labor, it seems to me that the woman gets calmer and more confident in her ability to deliver” (Basso 2010:394)  “I sort of can’t wait to have my first child… What an experience”, “It was shown not as a medical phenomenon, but a spiritual and emotional one” (Cleeton,2001:108) |  |
| Want information tailored to personal circumstance |  |  |  |  |
| Desire to learn from other women's lived experiences |  |  |  |  |
| Like interactive interventions | Women want multiple modes and formats of educational interventions with different women having different levels of literacy, comprehension or requisite skills and access to resources | 36,37,38,39,40,41,46 | "I find that’s very clear … the number format. The figure format, that wouldn’t be the way I would choose to view it … and probably not the pie chart format either", "[I liked] the pie charts … If you see 2 in a 100 you think oooh, but on the grand scale of a pie chart, you think, oh yeah it is small" (Emmett 2007:168).  “I would really like to know if I could have that book” (Shorten 2015;398).  “I just get nervous about who has that information . . . if they have a privacy thing on the Web site or knowing that your information will not be shared with someone else.” (Shorten 2015:394) |  |
| Still desire hard copies of information |  |  |  |  |
| Need to revisit information (at multiple time points and with multiple discussants) |  |  |  |  |
| Desire for emotional support | Women desire emotional support alongside the communication of facts and figures about birth | 36,38,39,40,41,43,45 | “This time my husband participated this program and he also often studied this internet course and we had the same concepts after discussion” (Wang 2006:6)  “Emotionally, it’s just been a closed door.” (Farnworth, 2008:120)  “If she had been more humane and listened more, I think I would have responded differently.” (Ramvi 2010:271)  “The doulas seem like they would be so much more comforting.” (Cleeton 2001:198) |  |
| Influence of previous life/birth experience | Women welcome health professional’s acknowledgement of previous birth (and life) experiences as an important component in decision-making about future birth method | 36,37,38,40,41,42,43,44,45,47 | “…Very, particularly with me having had such a traumatic first birth, the fears that I’m taking into this one” (Frost 2009: 900)  "I felt robbed of pushing my baby out myself" (Shorten 2004)  “I do think it’s important because having a caesarean birth, especially if it’s not planned, it, . . . it’s like a massive blow, so it, I felt sort of, it felt good to talk about it” (Farnworth 2008:120)  “When I tried to explain my situation, I was not heard.” (Ramvi 2010:272) | Interactions with health professionals and influence of healthcare system |
| Belief in value and significance of vaginal birth |  |  |  |  |
| Fear of pain and childbirth |  |  |  |  |
| Need for additional dialogue with health professionals | Intervention content as most useful when it complements clinical care, is consistent with advice from health professionals and provides a basis for more informed, meaningful dialogue between women and care providers | 36,37,40,41,43,45,46,47 | “I’ve kind of gone [to Obstetrician] with questions… in order to ask, sort of their medical opinion and their experience and what’s best to do…” (Frost 2009:899)  ‘I came out of there [the 36 week appointment] more confused” (Farnworth 2008:121)  “Basically, what it says there is what the doctors had said or what I’d been through… But seeing it there, it makes you understand better, because when a doctors telling you, you’re in, sort of shock.” (Emmett, 2007:167)  “The booklet was very easy to understand and has made me think more seriously about the options available to me. As a result of completing your survey I have put my fears and worries on paper and will make sure that my birthing options are discussed with my doctor…” (Shorten 2004:311) |  |
| Need consistent, credible information about either birth method |  |  |  |  |
| Desire to be involved in decision-making | Women’s attitudes towards involvement in decision-making vary | 37,38,40,41, 43,44,45,46, 47 | “We intend to talk a lot with the team. Negotiate.” (Basso 2010:396)  “I’m not sure that there will be any decisions that I can be involved in. When it comes to something like this…I don’t have enough knowledge, medical knowledge to say anything” (Frost 2009:899) |  |
| Uncertainty of role in decision-making |  |  |  |  |
| Reluctance to be involved in decision-making |  |  |  |  |
| Belief hidden agenda: manage information to encourage particular birth method | Women are aware of how the organisation of care and information impacts on the actual choices available to them | 36,37,38,39,40,41,43,44,  45,47 | “It’s very much biased towards ‘let’s have a vaginal delivery this time.’ I think if I’d wanted a section, I’m not sure you’d have a fight on your hands but you’d have to be strong.” (Farnworth 2008:121)  “I was terrified of getting, it sounds awful, but a doctor that was going to try and push me into their way of thinking… and I have to say I was pleased I didn’t come up against that.” (Farnworth 2008:120)  “How do I know, as a concerned father/husband, when is it good to have a C-section? Can we trust medical professionals? My mother had one and so did my step-mom-and neither have any apparent negative feelings towards it.” (Cleeton 2001:196)  “You know, what I’m saying, [women] that really don’t need to be cut on and some of them do. The procedure should be more - You know, the paperwork, they should follow them more to see what they’re doing.” (McCants 2015:132) |  |
| Expectation of a fight in pursuit of real choice |  |  |  |  |
| Clinical responsibility and accountability |  |  |  |  |
