# Supplementary material for: Women’s and communities’ views of targeted educational interventions to reduce unnecessary caesarean section: a qualitative evidence synthesis
Source: Reprod Health. 2018 Jul 24;15:130. doi: 10.1186/s12978-018-0570-z (PMC6057083; doi:10.1186/s12978-018-0570-z)
Supplement: Supplementary file 6 — Table S2. CERQual Summary of evidence profile table. (DOCX 33 kb) [file 12978_2018_570_MOESM6_ESM.docx]

**Additional Information (Table 2: CERQual Summary of evidence profile table)**

| **Review finding** | **Contributing studies** | Methodological Limitations | Coherence | Adequacy | Relevance | ***Assessment confidence in the evidence*** | ***Explanation of confidence in the evidence assessment*** |
| --- | --- | --- | --- | --- | --- | --- | --- |
| ***Theme 1: Mutability of women’s and community’s beliefs about birth: Ambivalence, Empowerment and Fear*** | | | | | | | |
| **Women and communities liked learning new information about birth:** The content and design of interventions opened up new ways of thinking about vaginal and caesarean birth for women and communities. Women described how educational interventions informed them about risks and benefits of vaginal birth and caesarean section that were hitherto unknown. Some women were surprised by the actual number of caesareans performed and the risks associated with them. Interventions brought issues of risk to the fore and forced pregnant women in particular to think through more clearly what mattered to them. | 36,37,38,39,40,41,42,43,44,45,46,47 | Minor concerns regarding methodological limitations in 5 of 12 studies. | Minor concerns about coherence as several studies also found new information increased anxiety. | No or very minor concerns regarding adequacy with rich data, from all 12 included studies. | Moderate concerns regarding relevance as 11 studies from high-income countries. | High confidence high-income countries and **moderate confidence** for whole review population. | 12 studies with minor methodological limitations. Rich data from 7 countries across 3 geographical regions with highest rates of unnecessary caesarean section. No or very minor concerns about coherence. |
| **Women described pregnancy as an inherent time of uncertainty and transformation of thought about birth method:** While some women described being very sure about their preferred method of delivery prior to or early in the current pregnancy, many of these same women later changed their minds following experience of an intervention or in response to evolving circumstance. Educational interventions played an important role in helping women prepare for any eventually and to reconcile the benefits of their actual birth method when it did not correspond to their preference. | 37,40,41,42,44,45,47 | Minor methodological limitations in 2 of 5 studies. | No or very minor concerns regarding coherence. Data similar within and across studies. | Moderate concerns regarding adequacy with rich data from Westernised settings (Europe and the Americas). | Moderate concerns regarding relevance as 5 studies from high-income countries. | Moderate confidence | 7 studies, 4 of which had very minor methodological limitations. Data from Europe and the Americas only. Very minor concerns about coherence. |
| **Communication of new knowledge and/or support can be empowering:** Learning risks were small, or what labour actually entails, enabled some women to feel more prepared and more confident to labour, especially were information about physiological processes was combined with emotional support. Pregnant or recently delivered women described how they had used information to gain control in the pursuit of informed decision-making; either by using the information to inform more meaningful dialogue with health professionals, or to justify a pre-existing preference for either birth method. | 36,37,38,39,40,41,42,44, 45 | Minor concerns regarding methodological limitations in 3 of 9 studies. | Minor concerns regarding coherence as review finding cogent with the different ways women described interventions gave them new confidence and control. But not all women felt this. | Minor concerns regarding adequacy with sufficiently rich data, from 9 of 12 included studies. | Moderate concerns regarding relevance as 8 studies from high-income countries. | Moderate confidence | 9 studies with minor methodological limitations. Sufficiently rich data from 7 countries across 3 geographical regions with highest rates of unnecessary caesarean section. No or very minor concerns about coherence. |
| **Some information can provoke fear:** Some women and communities found intervention content alarming. Childbirth education video content was described as too gory by a few nulliparous students. Some pregnant women said the use of computer or DVD decision-aids for VBAC increased their anxiety. Use of a decision-aid, combined with follow-up by a midwife helped mediate pregnant women's concerns about risk in one study, while midwives failing to listen to women's concerns and forcing them to birth vaginally compounded fears in another. | 36,38,39,40,41,45,47 | Minor concerns regarding methodological limitations in 2 of 7 studies. | Minor concerns regarding coherence as fear and anxiety is reported at different levels of interpretation across different studies. | Moderate concerns regarding adequacy with fairly rich data from 7 of 12 studies. | Moderate concerns regarding relevance as all 7 studies from high-income countries. | Moderate confidence | 7 studies with minor methodological limitations. Fairly rich data from USA, UK, Taiwan and Norway. Minor concerns about coherence. |
| **Theme 2: Multiplicity of birth information needs: Framing, format and individual management strategies** | | | | | | | |
| **Targeted educational interventions are only one component informing women’s and communities’ views and decision-making about birth method:** Women describe being exposed to a multiplicity of information sources in their pre-, present- and post-pregnancy trajectories. Some women using decision-aids describe them as “a starting point”; a springboard for seeking more information. Learning from the birth stories of family and friends was widespread. Information was also actively sought in the media and from the Internet, while face-to-face interactions with health professionals were viewed as the most important influence on actual birth method. | 36,37,38,40,41,42,43,44,45,47 | Minor concerns regarding methodological limitations in 5 of 10 studies. | Minor concerns regarding coherence with studies demonstrating how multiple sources of information impact how women perceive the benefits of an intervention. | Minor concerns regarding adequacy with sufficiently rich data from 10 of 12 studies. | Moderate concerns regarding relevance as 9 studies from high-income countries. | Moderate confidence | 10 studies with minor methodological limitations. Fairly rich data 7 countries across 3 geographical regions with highest rates of unnecessary caesarean section. No or very minor concerns about coherence with the other 2 studies not attending to this issue. |
| **Desire for educational content conveying the physical work of labour and the social and emotional impact of vaginal and caesarean birth for women.** Across settings and education formats, women and communities offered suggestions of what was missing from interventions. They wanted to know more about VBAC and homebirth, what a midwife does, maternity entitlements, the social and emotional impact of caesarean birth, and the “body work” vaginal birth entails. Women also felt vaginal birth could be presented in a more positive way by acknowledging it as an experience. They also wanted information framed in ways women could more easily relate to; for example, many women desired to learn about birth from other women’s experiences; some women wanted information about interventions that was personalised. | 36,37,39,40,41,42,43,44,45,46,47 | Minor methodological limitations in 4 of 11 studies | No or very minor concerns regarding coherence. Data similar within and across studies. | Minor concerns regarding adequacy with rich data from a range of settings, contexts and sub-populations | Moderate concerns regarding relevance as 10 studies from high-income countries. | High confidence high-income countries and **moderate confidence** for whole review population | 11 studies with minor methodological limitations. Data from 6 countries across 3 geographical regions. Very minor concerns about coherence. |
| **Women want multiple modes and formats of educational interventions:** Women and communities had wide-ranging views on appropriate language use, figures and tables to communicate information across formats. While many could see the benefits of computer-based interventions, ease of use was problematic for some and pregnant women in particular still desired hard copies of information to revisit and discuss with family members and healthcare professionals. Some concern was expressed about the confidentially of information in on-line decision-aids. Video content was largely welcomed as it facilitated the visualisation of positive, actual birth experiences. | 36,37,38,39,40,41,46 | Minor concerns regarding methodological limitations in 1 of 5 studies. | Moderate concerns about coherence as contributory data is largely limited to the level of description. Data similar across studies. | Moderate concerns regarding adequacy with rich data from 5 of 12 studies. | Moderate concerns regarding relevance as all 5 studies from high-income countries. | Moderate confidence | 7 studies with minor methodological limitations. Data from 3 countries (UK, USA, Taiwan) across three geographical regions. Moderate concerns about adequacy and coherence. |
| **Women desired emotional support alongside the communication of facts and figures about childbirth:** Women perceived the choice between vaginal birth and caesarean section as huge; with far-reaching consequences for health and wellbeing. Pregnant women in particular described needing emotional support alongside information about the risks and benefits of birth methods. In tandem with interventions women described additional emotional support from husbands, health professionals and doulas. | 36,38,39,40,41,43,45 | Minor concerns regarding methodological limitations in 2 of 7 studies. | No or very minor concerns about coherence. Data similar within and across studies. | Moderate concerns regarding adequacy with fairly rich data from 7 of 12 studies. | Moderate concerns regarding relevance as all 7 studies from high-income countries. | Moderate confidence | 7 studies with minor methodological limitations. Fairly rich data from 5 countries (UK, USA, Australia, Norway and Taiwan). 6 of the 7 studies involved pregnant or post-natal women faced with the gravity of the actual decision-made. |
| ***Theme 3: Interactions with health professionals and influence of healthcare system: Support, consistency and autonomy*** | | | | | | | |
| **Women welcome health professional’s acknowledgement of previous birth (or life) experience as an important step in decision-making about future birth method.** While previous experiences are important in attitude formation they do not necessarily equate to subsequent preference for delivery method. Across study settings many women and communities valued vaginal birth as natural and a meaningful life experience for women, with fears associated with labour and vaginal birth (pain, uterine rupture) not insurmountable. Few women categorically preferred caesarean section. Some women who had previous experience of caesarean section were particularly keen to avoid it. | 36,37,38,40,41,42,43,44,45,47 | Minor concerns regarding methodological limitations in 5 of 10 studies. | No or very minor concerns regarding coherence. Data similar within and across studies. | Minor concerns regarding adequacy with data from 10 of 12 studies; richest data from European settings. | Moderate concerns regarding relevance as 9 studies from high-income countries. | Moderate confidence | 10 studies with minor methodological limitations. Data from 7 countries across 3 geographical regions with richest data from European settings. Minor concerns about coherence. |
| **Intervention content was most useful when it complemented clinical care, was consistent with advice from health professionals and provided a basis for more informed, meaningful dialogue between women and care providers**: Some women and communities experiences of interventions suggest they raised more questions than they answered and created a need for additional dialogue with health professionals to discuss issues raised, fears evoked, and revisit birth plans. While some pregnant women described themselves as "desperate" for such conversations, other women were dissatisfied when their expectations went unmet because conversations were too brief, their views were not listened too, the health professional was unknown to them, and/or gave inconsistent advice. | 36,37,40,41,43,45,46,47 | Minor concerns regarding methodological limitations in 3 of 8 studies. | No or very minor concerns regarding coherence. Data similar across studies. | Minor concerns regarding adequacy with sufficiently rich data from 8 of 12 studies. | Moderate concerns regarding relevance as 8 studies from high-income countries. | Moderate confidence | 8 studies with minor methodological limitations. Data from 4 countries across 3 geographical regions. No or very minor concerns about coherence. |
| **Women’s attitude towards involvement in decision-making:** Some women have a strong desire to be involved and to exert control in the decision-making process; others are less certain of their role and value some involvement; while others still are reluctant for any involvement and want qualified health professionals to make the decision for them. The success of any intervention to reduce unnecessary caesarean section is dependent upon pregnant women being open to a role in decision-making and some degree of uncertainty surrounding preference for caesarean section. | 37,38,40,41,43,44,45,46,47 | Minor concerns regarding methodological limitations in 4 of 9 studies. | Minor concerns about coherence. Data similar within and across studies, only variation in degree of involvement women report. | Minor concerns regarding adequacy with rich data from 10 of 12 studies. | Moderate concerns regarding relevance as 8 studies from high-income countries. | Moderate confidence | 9 studies with minor methodological limitations. Data from 7 countries across 3 geographical regions. Minor concerns about coherence. |
| **Women are aware of how the organisation of care and information impacts the actual choices available to them:** Some women and communities believed intervention content favoured health professionals’ hidden agendas to promote whichever method of birth was favoured by them or the hospital or health system in which they work. In two geographical regions pregnant women used metaphors of conflict in the pursuit of their choice of birth method. Other women questioned the exclusion of information about homebirth, excessively high caesarean section rates, and why doctors aren't publically accountable for the number of caesarean sections performed if they are "cutting on women" unnecessarily. | 36,37,38,39,40,41,43,44,  45,47 | Minor concerns regarding methodological limitations in 4 of 10 studies. | Moderate concerns about coherence as insufficient exploration of suspicion in contributory studies. Data strikingly similar across studies. | Minor concerns regarding adequacy with fairly rich data from 10 of 12 studies. | Moderate concerns regarding relevance as 9 studies from high-income countries. | Moderate confidence | 10 studies with minor methodological limitations. Data from 7 countries across 3 geographical regions. Moderate concerns about coherence. |
